# Modulation of neuronal entrainability by epilepsy-associated currents and noise: a spectral approach

**DOI:** 10.1186/1471-2202-15-S1-P202

**Published:** 2014-07-21

**Authors:** Alla Borisyuk

**Affiliations:** 1 Department of Mathematics, University of Utah, Salt Lake City, UT 84112, USA

## 

During an epileptic seizure a wave of population activity is moving through neural tissue. To understand its development it is crucial to know whether or not the neurons are going to be recruited into the wave. We derive an indirect indication for how easy it would be for the neurons to be recruited, by estimating their entrainment ability to periodic pulsatile stimulus. We are especially interested to know how the entrainment ability is modified when intrinsic properties of the cell are altered, particularly properties of the currents that have been implicated in certain kinds of epilepsy.

In this study we investigate the effects of periodic stimulation on two (noisy) neuron models: including an M-type potassium and a hyperpolarizing cation (H-) current, respectively. The (over/under)-expression of these channels has been associated with epileptogenesis [1]. Varying the maximal conductances of the M- and H-currents qualitatively changes the shape and magnitude of the model neurons’ phase resetting curves (PRCs) [2].

We begin by representing the neurons by their noisy phase resetting curves (PRCs), where the PRCs’ variances are computed according to [3]. From these data we create stochastic phase map, taking careful account of the possibility of multiple inputs per cycle. The stochastic phase map takes phase of a spike relative to the periodic stimulus and maps it to the phase of the subsequent spike. Next, we compute and analyze the spectrum of the Markov transition operator for this stochastic circle map. Pathwise dynamic properties, such as stochastic periodicity (phase-locking), stochastic quasi-periodicity, and chaotic behavior, can be distinguished using the geometry of the transition operator’s spectrum [4]. As a result, a measure of neuron's ability to entrain is obtained. Notably, this entrainability measure, unlike commonly used vector strength is computed from the operator, without the need to resort to direct simulations.

The procedure is repeated for a number of parameter combinations (M- or H- current maximal conductance, noise level, stimulus strength, stimulus frequency) to explore the dependence of the phase maps’ spectral properties (and resulting entrainability) on intrinsic and input parameters.
